# Excited State Dynamics of Alizarin Red S Nanoparticles in Solution

**DOI:** 10.3390/molecules28155633

**Published:** 2023-07-25

**Authors:** Pascal Rauthe, Kristina Sabljo, Maike Kristin Vogelbacher, Claus Feldmann, Andreas-Neil Unterreiner

**Affiliations:** 1 Institute of Physical Chemistry, Karlsruhe Institute of Technology, 76131 Karlsruhe, Germany; 2 Institute of Inorganic Chemistry, Karlsruhe Institute of Technology, 76131 Karlsruhe, Germany

**Keywords:** alizarin red S, nanoparticles, aqueous solution, femtosecond, pH dependence

## Abstract

Alizarin red S is a sulfonated, water-soluble derivative of alizarin. This work presents femtosecond studies of alizarin red S (ARS) nanoparticles in comparison to ARS in aqueous solution and to alizarin in DMSO. The femtosecond studies cover a probing spectral range of 350–750 nm using different excitation wavelengths, taking into account the variation of the absorption spectra with the pH values of the solvent. Stationary absorption spectra show slight differences between solution and nanoparticles. Excitation at 530 nm results in low and noisy responses, therefore, we additionally recorded transient spectra of the nanoparticles at λ_ex_ = 267 nm. While the results in DMSO are comparable to previous studies in non-aqueous solvents, we report a relatively fast relaxation of 14 ps in [La(OH)_2_][ARS] nanoparticles in aqueous solution after excitation at 530 nm, which is similar to Na(ARS) solution (19 ps). The dynamics changed with lower pH, but still without significant differences between nanoparticles and solution. We propose [La(OH)_2_][ARS] nanoparticles as a suitable alternative to dissolved molecules with similar spectroscopic properties, for example, with regard to biomarker applications.

## 1. Introduction

Alizarin and its derivatives have been the subject of many studies due to their interesting photophysical properties [1,2,3]. The additional sulfonate group in alizarin red S (ARS) allows for the investigation of photophysical properties in aqueous solution. Due to its spectroscopic properties, with strong absorption and low fluorescence, there is a potential for use in biological systems [4]. In general, ARS and alizarin do not show many differences in their spectroscopic behavior, however, when it comes to biological applications, solubility in water is a crucial factor. Alizarin is not soluble in water; therefore, ARS is the better choice in this case. Recently, nanoparticles have been developed to target interest in their functionality as biomarkers [5] and even as nanocontainers for selective drug delivery [6,7]. Aiming at drug delivery and multimodal imaging, we have developed the concept of inorganic–organic hybrid nanoparticles (IOH-NPs) [7,8]. A previously published article describes the synthesis and stationary spectroscopic behavior of saline [La(OH)_2_][ARS] nanoparticles, which are composed of [La(OH)_2_]^+^ as the cation and [ARS]^−^ as the anion [9]. Some advantages of these [La(OH)_2_][ARS] nanoparticles compared to dissolved molecules are the high dye load (ARS with 65% of total nanoparticle mass), a higher local brightness, photostability, and sensitivity. They also offer better opportunities for functionalization [9]. Femtosecond studies help in understanding photophysical and chemical processes, in general. Alizarin has been extensively investigated in femtosecond studies, mainly for excited state intramolecular proton transfer (ESIPT) [10] and electron transfer (ET) [11,12,13,14]. However, ARS has been largely disregarded in this context. Alizarin’s and ARS’s ability to stain bone marrow has been known for over 100 years [15]. This is due to their ability to form chelate complexes with calcium [3]. Aiming at a modified delivery, such as the change from solvated molecules to dispersed nanoparticles, requires detailed clarification of the differences in (electronic) properties. Ideally, the differences in spectroscopic properties would be small when changing from dissolved molecules to solid nanoparticles. Despite the relatively low fluorescence quantum yield, the binding properties to metals and anions show a great potential in diagnostic research [16,17]. The selective affinity of drugs for cells has become a very important factor in medical diagnostics, but also in terms of treatment [18,19,20]. Understanding the excited state population and subsequent relaxation pathways can help to determine the reactivity and photodynamics of substances for potential applications [21]. 

## 2. Results and Discussion

### 2.1. Nanoparticle Synthesis

**Figure 1:**
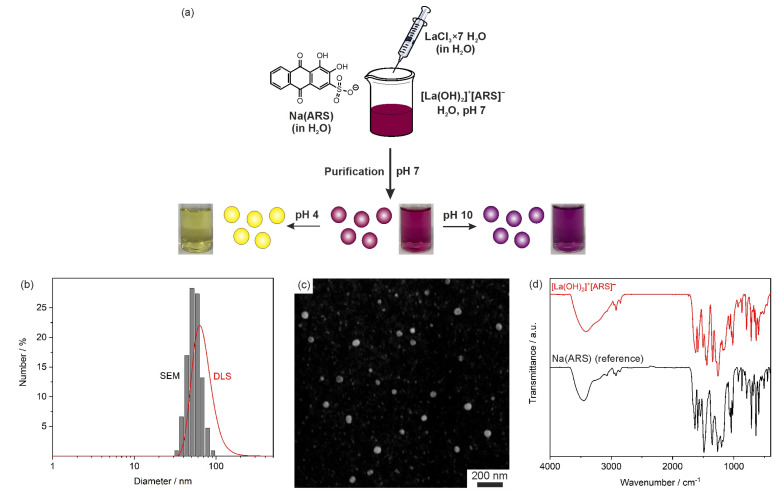
Characterization of [La(OH)_2_]^+^[ARS]^−^ IOH-NPs: (**a**) Scheme illustrating the aqueous synthesis of [La(OH)_2_]^+^[ARS]^−^ IOH-NPs with photos showing the color of suspensions at different pH values; (**b**) Particle size distribution according to DLS (in water) and statistical evaluation of >100 particles on SEM images; (**c**) SEM image; (**d**) FT-IR spectrum (Na(ARS) as a reference) (modified illustration from reference [9]).

Essentially, the IOH-NP concept is characterized by saline compounds, consisting of an inorganic cation and a functional organic anion. The functional organic anion can be a drug or fluorescent dye with phosphate, sulfonate, or carboxylate groups. Together with a suitable cation, the drug/dye anion forms an insoluble saline compound in water. IOH-NPs are characterized by simple aqueous synthesis and an unprecedented load of drug/dye (70–85 wt% of total nanoparticle weight). [La(OH)_2_]^+^[ARS]^−^ is a typical example of such IOH-NPs with lanthanum (La^3+^) as the inorganic cation and the pH indicator alizarin red S (1,2-dihydroxy-9,10-anthraquinonesulfonicacid, ARS) as pH-sensitive dye anion (Figure 1a) [9]. We already used [La(OH)_2_]^+^[ARS]^−^ IOH-NPs to determine the pH in vitro in cells based on the fluorescence of the IOH-NPs. Upon variation of the pH value, the [La(OH)_2_]^+^[ARS]^−^ IOH-NP suspensions show a pH-dependent color change (Figure 1a) as well as pH-dependent emission [9].

### 2.2. Stationary Absorption Spectra

**Figure 2:**
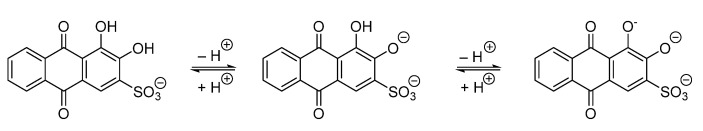
Differently charged forms of ARS. The sulfonate group is always deprotonated.

**Figure 3:**
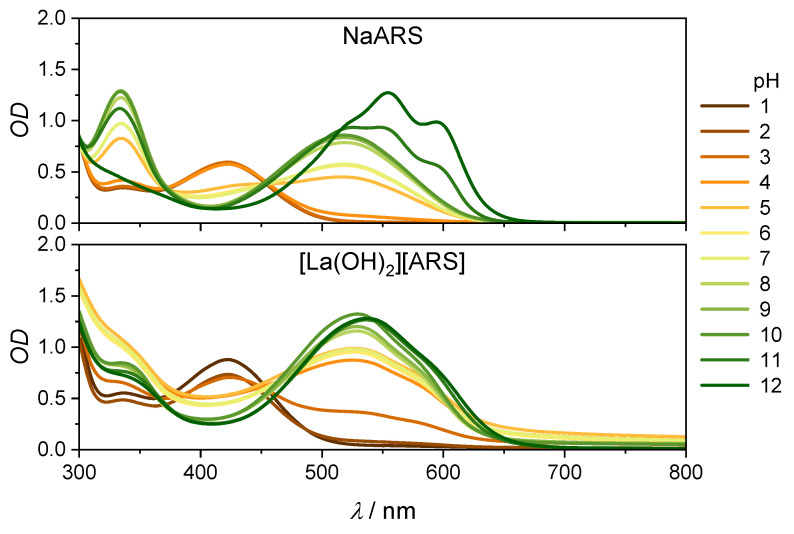
Stationary spectra of Na(ARS) (**top**) and [La(OH)_2_][ARS] nanoparticles (**bottom**) in water as a function pH. Path length 1 cm, c(ARS) = 0.137 mM. Scattering nanoparticles leads to an increased offset.

Experiments on the characterization and stability of the [La(OH)_2_][ARS] nanoparticles were previously published by Sabljo et al. [9] Accordingly, [La(OH)_2_][ARS] nanoparticles have a pH window of five to nine, in which where they are stable and have low solubility. The absorption spectra of ARS show a strong pH dependence (see Figure 2 and Figure 3). In the three different protonation stages, the color changes from yellow (protonated) to red (monoanion) to purple (dianion).

**Figure A1:**
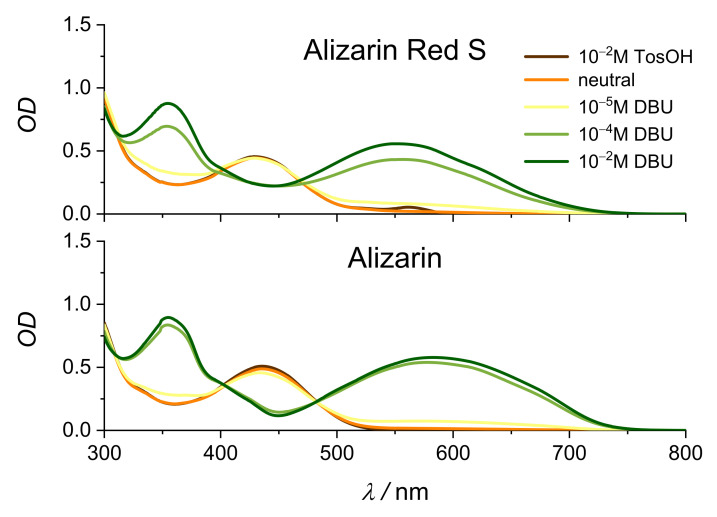
Stationary spectra of alizarin red S and alizarin in DMSO with different concentrations of toluenesulfonic acid (TosOH) and Diazabicycloundecene (DBU), respectively. Path length 1 mm, c(ARS/Alizarin) = 0.9 mM.

The protonation of the sulfonic group is of secondary relevance here. Sulfonic acids have low pK_a_-values [22]. Hence, protonation of this group is not possible in aqueous systems and does not matter in terms of modified protonated stages of ARS. Comparing alizarin with ARS is not possible in aqueous solution due to the lack of solubility of alizarin. Solvent mixtures have been attempted to circumvent this problem [2,10], but in this study, the pure solvent approach was preferred. Alizarin in DMSO was investigated by Jen et al. [23,24], including emission and transient spectra. Due to the good solubility of ARS in DMSO, we chose this as a solvent. Substituted basic components shift the absorption maximum from roughly 430 to 550 nm. Absorption spectra in DMSO (Figure A1) show the influence of the sulfonate group in ARS, which slightly shifts the absorption maximum from 436 to 430 nm and from 581 to 553 nm, respectively.

The addition of lanthanum ions induces a bathochromic shift in the absorption spectra of ARS solutions [25]. This effect is mainly due to the stabilization of the monoanionic form of ARS, which seems to be favored in the presence of La^3+^.

Changing the solvent from DMSO to water leads to hypsochromic shifts in both the protonated (430→422 nm, 441 cm^−1^) and monoanionic forms (553→518 nm, 1222 cm^−1^). Suzuki et al. [26] reported a pK_a_ = 5.33 for ARS; Shalaby et al. [27] reported pK_a_ = 5.82 for the first and pK_a_ = 10.78 for the second deprotonation. This is in agreement with the spectra in Figure 3. The [La(OH)_2_][ARS] nanoparticles dissolve around pH 4 and below [9]; the spectra at this pH are a mixture of solution and suspension. However, the presence of nanoparticles can be identified by the offset shift. The [La(OH)_2_][ARS] nanoparticles increase the offset of the absorption spectrum, which distorts the isosbestic points. Another notable difference is the development of the two absorption bands at pH > 10. The second deprotonation seems to be hindered by the [La(OH)_2_][ARS] nanoparticles, a characteristic formation of the two absorption maxima, which is by far not as strong as in the dissolved ARS. 

**Figure 4:**
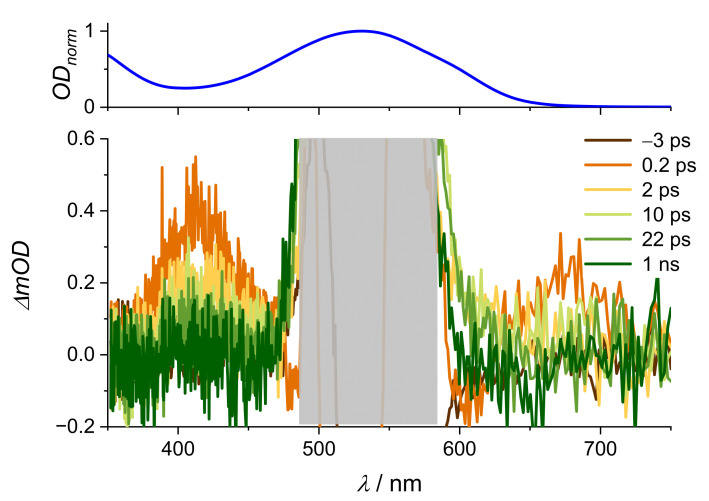
(**Top**): stationary absorption spectrum and (**bottom**): transient absorption spectra of [La(OH)_2_][ARS] nanoparticles in H_2_O (pH = 7), OD_400nm_ = 2.13, λ_ex_ = 530 nm, E = 0.9 µJ at delay times as indicated (ps = picosecond, ns = nanosecond). Grey bar covers the scattered light from the excitation pulse.

**Figure A3:**
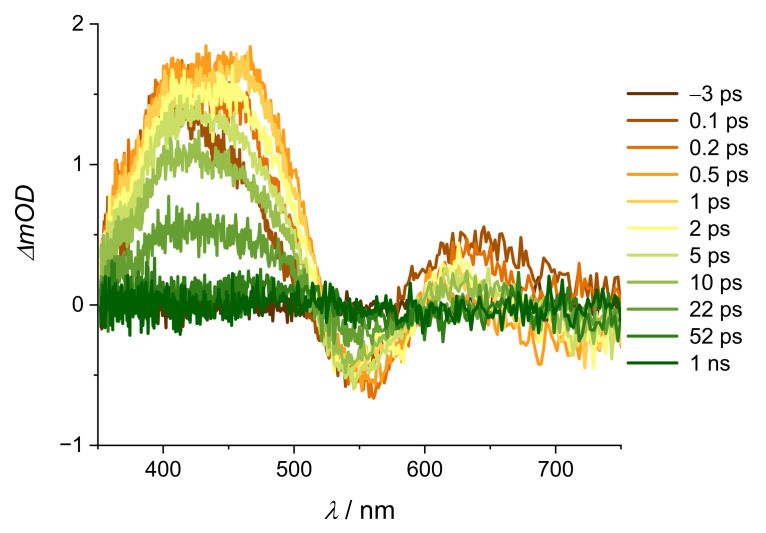
Transient absorption spectra of Na(ARS) in water (pH = 7), OD_267nm_ = 2.38, λ_ex_ = 267 nm, and E = 0.5 µJ.

Acidic forms of alizarin derivatives have been well investigated, but since the [La(OH)_2_][ARS] nanoparticles are mostly stable at pH 5–9, we will first focus on pump-probe experiments under these conditions. Lanthanum-based alizarin compounds and [La(OH)_2_][ARS] nanoparticles have not, to the best of our knowledge, been studied on femtosecond timescales, but ET studies with alizarin on TiO_2_ have been quite well examined [11,14,28,29]. The presence of TiO_2_ shifts the absorption spectrum of alizarin from 430 to approximately 500 nm [11]. According to the absorption spectra in Figure 3, TiO_2_ deprotonates alizarin to the monoanionic form, which is the same effect that occurs with La^3+^ [25]. These results with TiO_2_-alizarin nanoparticles may indicate that the [La(OH)_2_][ARS] nanoparticles also contain an ET, which should not occur in solution. The nanoparticles scatter more than the solution of the irradiation pulse instead of absorbing it, resulting in a weak signal. Figure 4 shows the spectra of the nanoparticles and respective results for the aqueous ARS-solution are shown in Figure A3.

**Figure 5:**
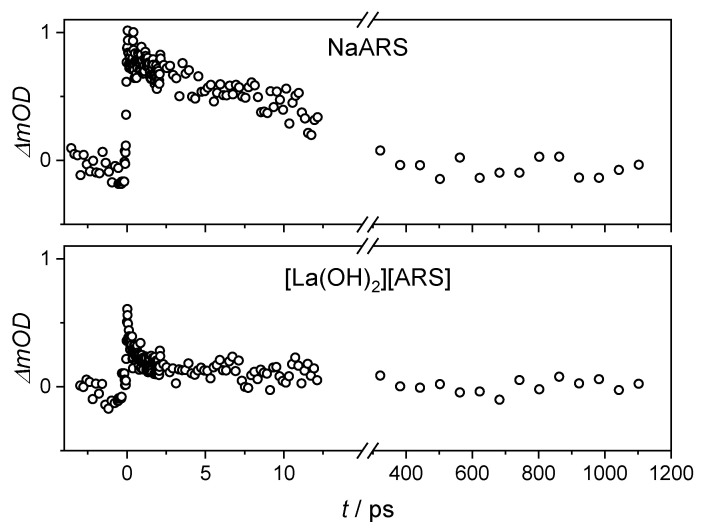
Single transients at a probe wavelength of 410 nm for aqueous Na(ARS) solution and [La(OH)_2_][ARS] nanoparticles with λ_ex_ = 530 nm and E = 0.9 µJ.

The intensity was surprisingly low in both solution and suspension, most likely due to a larger beam diameter of the 530 nm excitation pulse compared to 400 nm. The excited state lifetime was roughly 19 ps in the solution and 14 ps in the [La(OH)_2_][ARS] nanoparticles (Table 1), which is half the lifetime of the ESA in an acidic environment. The negative response in at 0.2 ps is most likely due to chirp contribution (Figure 5).

Even at low intensities, it can be shown that dissolved ARS and [La(OH)_2_][ARS] nanoparticles still show a similar relaxation. The similar results show that the optical properties of ARS are more or less retained independent of simple dissolution or attached to nanoparticles. Only the scattering increases, and the second time constant in the dynamics changes slightly. This proves that nanoparticles are equivalent to dissolved species in their function as spectroscopic markers but add new properties with their composition. Considering the publications on ET in TiO_2_-alizarin, it is likely that τ_1_ in the [La(OH)_2_][ARS] nanoparticles comprises ET, while τ_1_ in solution is the response of a vibrational cooling. The ET in TiO_2_-alizarin nanoparticles has been published with τ~100 fs [14,29] after excitation at 529 nm, so 260 fs in [La(OH)_2_][ARS] nanoparticles seems reasonable. Still, the accordance in τ_2_ indicates that ARS does not lose its original main relaxation channel in the excited state and is still valid for practical applications. Certainly, quantitative conclusions are difficult with weak intensities, but the data still provide enough information for this conclusion.

**Figure 6:**
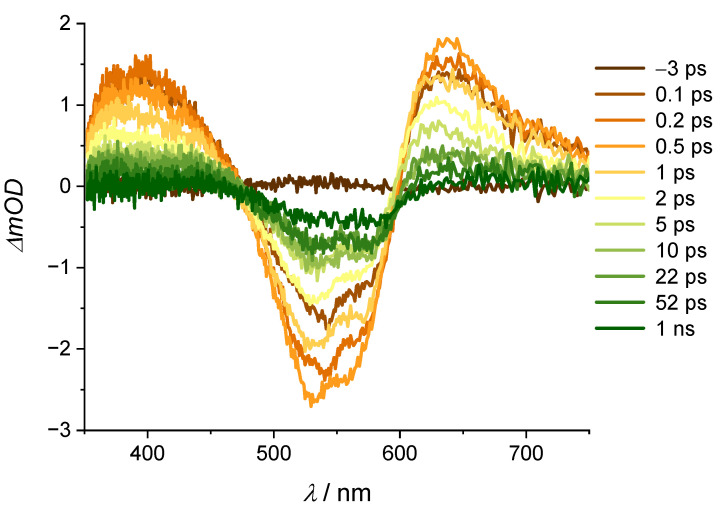
Transient absorption spectra of [La(OH)_2_][ARS] nanoparticles in water (pH = 7), OD_267nm_ = 2.33, λ_ex_ = 267 nm, and E = 0.5 µJ. (ps = picosecond, ns = nanosecond).

So far, we have shown transient spectra with excitation near the absorption maximum. This leads to a lot of scattered light from the excitation pulse on the detection CCD-camera, especially when [La(OH)_2_][ARS] nanoparticles are involved. It is possible to avoid this problem by switching the excitation pulse to a wavelength that is not within the detection window of the probing white light continuum, such as the third harmonic (267 nm) of the fundamental pulse. The excitation energy in these spectra is lower due to the increased OD in the stationary absorption spectra. Figure 6 shows the corresponding transient spectra.

Without the perturbations from the excitation pulse, three different transient bands are resolved in both spectra with some differences in the shape. The characteristic ESA that appeared after excitation at 530 nm is now much better resolved. All excited state dynamics in Figure 6 are fully recovered in the ground state within 50 ps, but the [La(OH)_2_][ARS] nanoparticles still show a slight response after 1 ns. With isosbestic points around zero, both spectra most likely still show S_1_-dynamics, which allows a comparison with the spectra after excitation at 530 nm. Table 2 shows that the time constant of 19 ps is still present after excitation into higher singlet states. There is also a time constant with 6 ps. 

However, it is not possible to reproduce the 14 ps in the [La(OH)_2_][ARS] nanoparticles (compare Table 1 and Table 2). Nevertheless, the agreement in the 6–7 ps component in Table 2 proves the similarity in both ARS conformations. The identification of time constants can be a numerical problem, but channel branching and other numerous effects cannot be easily disentangled by algorithms. Overall, the spectra after excitation at 267 nm and 530 nm show some differences, but the main properties of ARS are still intact in the nanoparticles. The trend of both spectra is the same but [La(OH)_2_][ARS] nanoparticles show a much stronger GSB at 550 nm, presumably due to a higher extinction coefficient and a much more pronounced ESA at 630 nm. In addition, the isosbestic points for the nanoparticles at 470 and 595 nm are not at zero amplitude. This indicates that at least two different excited states involved. Huber et al. had similar results with alizarin + TiO_2_ nanoparticles and associated this with an ET state [13]. The response in our [La(OH)_2_][ARS] nanoparticles is even stronger than the combination of alizarin + TiO_2_, which is probably due to the affinity of La^3+^ for charge transfer processes [30,31]. 

**Figure 7:**
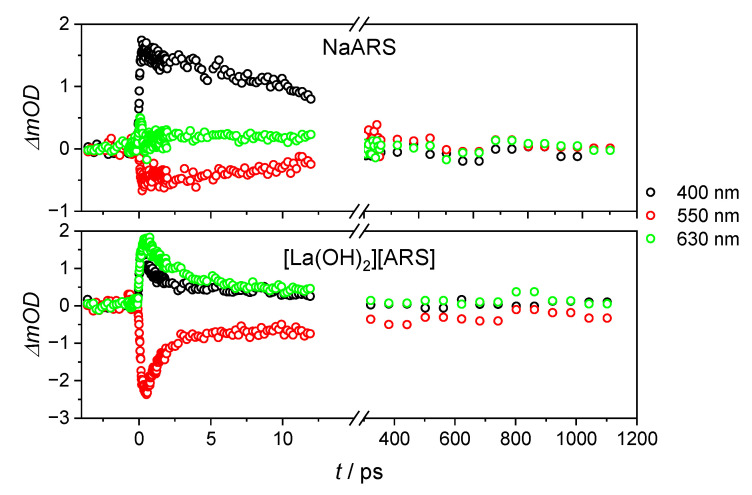
Single transient of aqueous Na(ARS) solution and [La(OH)_2_][ARS] nanoparticles at 400 nm with λ_ex_ = 267 nm and E = 0.5 µJ.

As with the single transients at λ_ex_ = 530 nm, the [La(OH)_2_][ARS] nanoparticles have an ultrafast component in the first 3 ps, which is present in the ARS solution (compare the black data points from Figure 5 and Figure 7).

**Figure A4:**
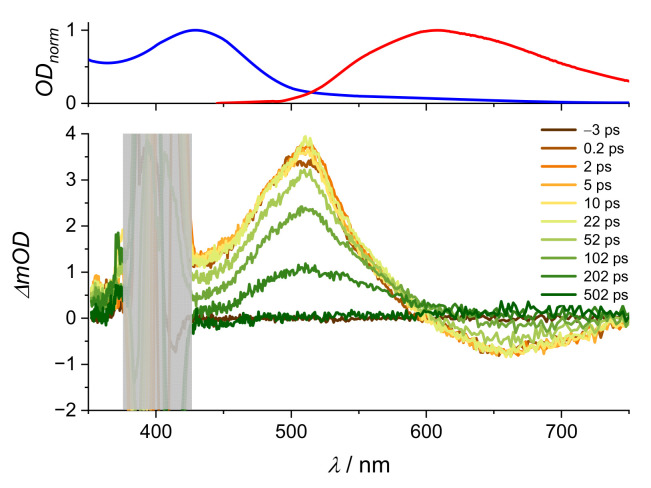
Transient absorption spectra of ARS^−^ solution in DMSO, OD_400nm_ = 0.39, λ_ex_ = 400 nm, and E = 1 µJ. Stationary absorption (blue) and emission spectra (red) are added above. Grey bar covers the scattered light from the excitation pulse.

**Figure A5:**
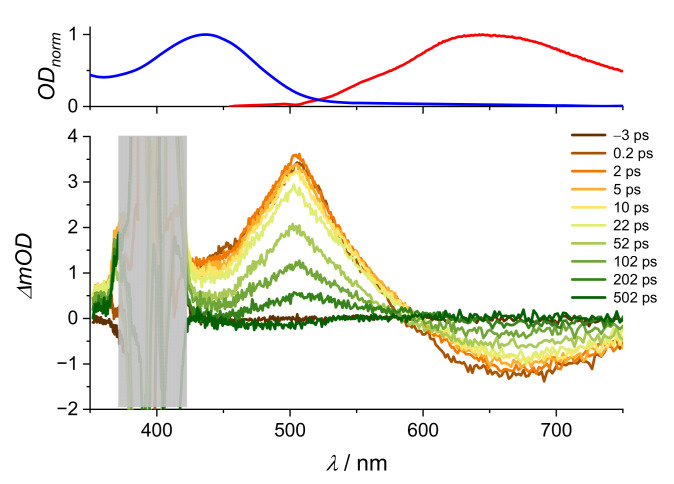
Transient absorption spectra of alizarin solution in DMSO, OD_400nm_ = 0.34, λ_ex_ = 400 nm, and E = 1 µJ. Stationary absorption (blue) and emission spectra (red) are added above. Grey bar covers the scattered light from the excitation pulse.

**Figure A6:**
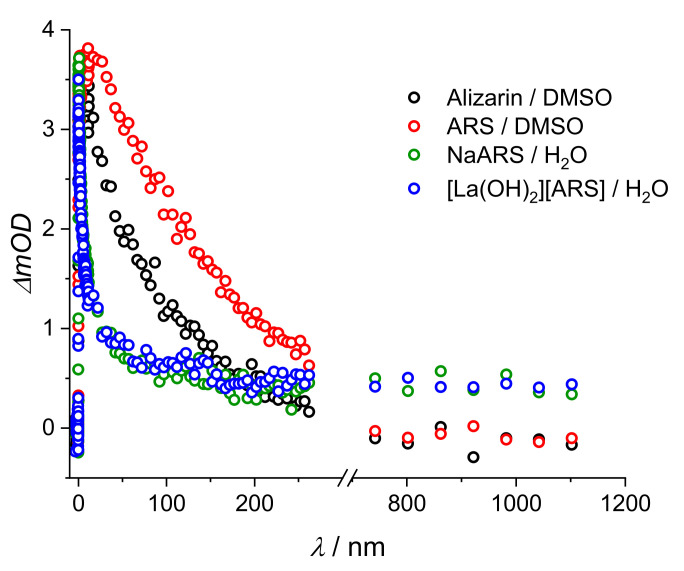
Single transients at 505 nm in respective solution.

In the past, alizarin derivatives have mainly been investigated in terms of the ESIPT, which occurs on a very short time scale. The ESIPT of alizarin in DMSO (110 fs) was studied by Jen et al. [23,24] via time-resolved Raman spectroscopy. Sulfonate has a positive Hammet parameter [32,33], therefore, the electron density is likely to increase in the aromatic system. This substitution has a massive effect on the transient lifetime (Figure A4, Figure A5 and Figure A6, Table A1). According to global fit results, the additional sulfonate group increases the excited state lifetime by about a factor of two from 89 to 169 ps. Interestingly, when only the stimulated emission (SE) is analyzed with the same method, the lifetime decreases from 84 (alizarin) to 62 ps (ARS). It is possible that the lifetime of the normal form increases while the tautomeric form lifetime decreases. We infer this from the fact that the SE around 660 nm originates only from the tautomeric form, whereas the ESA is a convolution of both. Quantum chemical calculations could support this assignment but were not part of this work. Ground state conformations and respective energies have been calculated [34] but, to our knowledge, excited state properties have only been calculated for alizarin [35,36].

**Figure 8:**
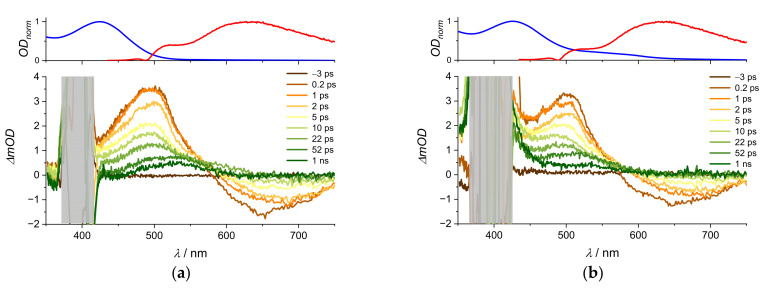
(**a**): Transient absorption spectra of Na(ARS) in H_2_O (pH = 3), OD_400nm_ = 0.54, (**b**): [La(OH)_2_][ARS] nanoparticles in H_2_O (pH = 3), OD_400nm_ = 0.57 (**bottom**). All samples were irradiated with λ = 400 and E = 1 µJ. The masked area in the vicinity of the pump pulse is broader for [La(OH)_2_] [ARS] due to higher scattering by the nanoparticles. Stationary absorption (blue) and emission spectra (red) are added on **top**. Grey bar covers the scattered light from the excitation pulse. (ps = picosecond, ns = nanosecond).

Aqueous solutions at pH 3 dissolve the [La(OH)_2_][ARS] nanoparticles [9], so Figure 8b most likely shows dissolved ARS. All spectra have one broad ESA band around 500 nm that shifts to 530 nm within 50 ps. However, all of the dynamics are recovered within half of a nanosecond in DMSO (see Appendix A Figure A4, Figure A5 and Figure A6), whereas there is still some ESA after one nanosecond in water. Interestingly, the ESA recovers completely at pH 1 (compare Figure A8 and Figure A9).

**Figure 9:**
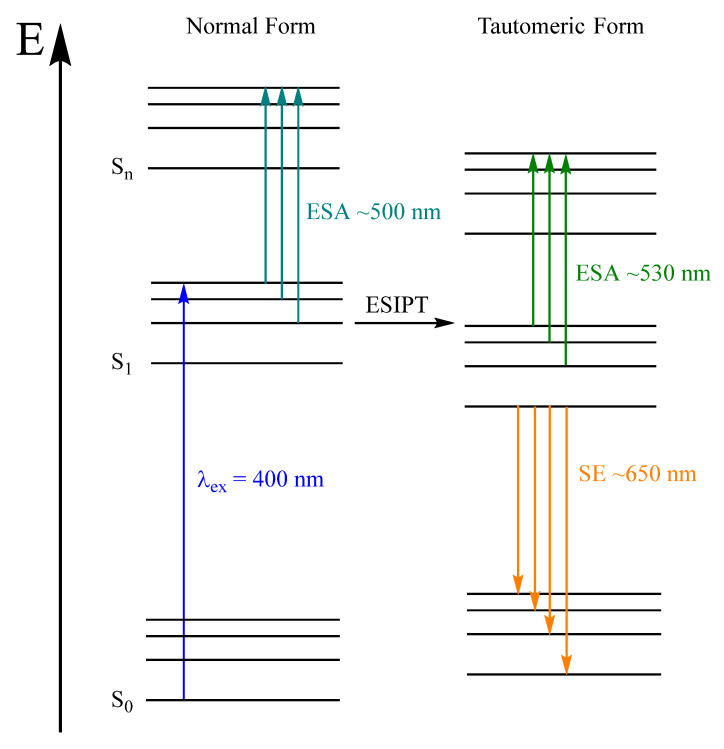
Jablonski diagram of acidic ARS after excitation with 400 nm. All observed processes are shown with colored arrows.

The ESIPT itself is not directly detected in the transient absorption spectra but the shifted ESA perfectly matches the absorption band of the proton transfer product, peaking around 530 nm. Consequently, we attribute this band to the tautomeric form. Typically, hot ground state absorption is red shifted with respect to the equilibrated system. In the present case, we do not observe any red shift other than the one responsible for the ESIPT. Theoretical calculations for alizarin support a red shifted tautomeric form [37]; according to our knowledge, there are no corresponding calculations for ARS. Instead, we added a Jablonski diagram to highlight the observed processes (Figure 9). Global fit time constants for the aqueous solution and [La(OH)_2_][ARS] nanoparticles are given in Table 3.

The first time constant is identical in both samples, which is consistent with vibrational energy redistribution and cooling in the excited state. Even when almost completely dissolved, the presence of [La(OH)_2_][ARS] nanoparticles increases the second time constant by a factor of two. Although this might be the influence of the few leftover nanoparticles, it could be also the presence of the La^3+^, which can affect the lifetime as well. Surprisingly, the results in Table 1 show a different trend by reducing the lifetime (19 to 14 ps) with nanoparticles instead of extending it (26.5 to 46.8 ps) The dual fluorescence in water indicates a strong competition between ESIPT and fluorescence of the normal form, which is significantly less pronounced in DMSO (see Figure A4 and Figure A5). From theoretical studies it has been concluded that the ESIPT lifetime increases with the polarity of the solvent [38,39]. Another factor may be the aproticity of DMSO. Contrary to this assumption, theoretical calculations predicted that alizarin and its derivatives are more likely to show an ESIPT in protic solvents [35], which drastically affects the excited state depending on the chosen solvent. Interestingly, the above-mentioned shift in water from 500 to 530 nm within 50 ps indicates the occurrence of a second excited state. A comparison of the spectra in Figure 8 with Figure A4 shows no shift in DMSO. As can be seen from Table A2, intensity-dependent measurements do not show evidence of singlet–singlet annihilation in the nanoparticles. While such a process has been proven to occur in nanoparticles [40], our study cannot completely rule it out because the intensity variation was limited to a factor of 2.5, due to stability issues of the samples and detection limitations.

**Figure A7:**
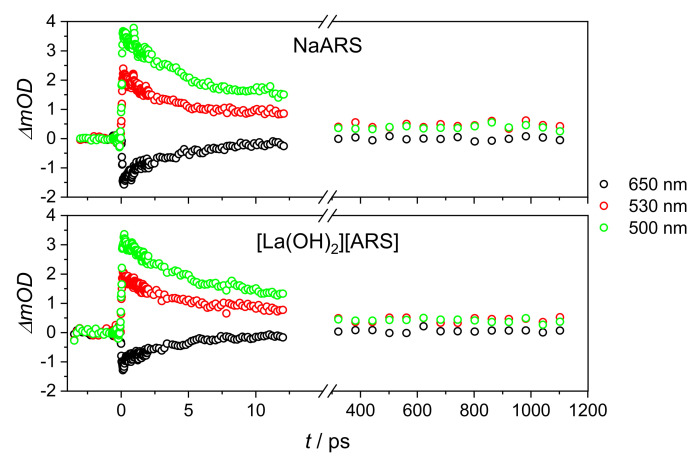
Single transients at probe wavelengths of 500, 530, and 650 nm in aqueous Na(ARS) solution (top) and [La(OH)_2_][ARS] nanoparticles (bottom).

**Figure A8:**
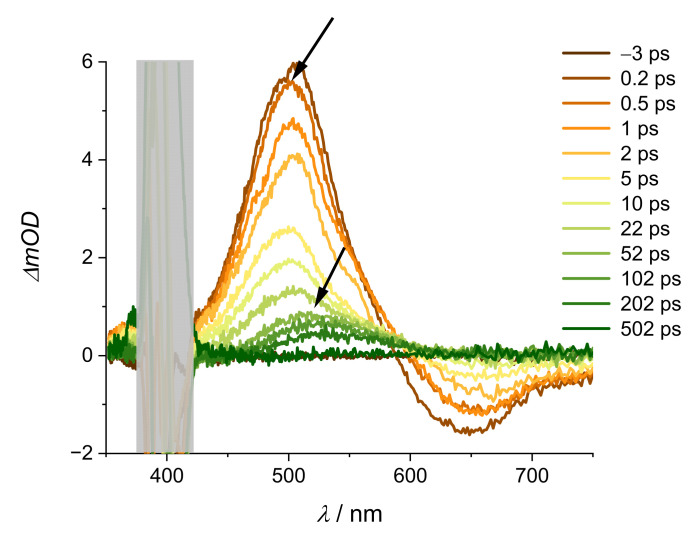
Transient absorption spectra of Na(ARS) solution in water (pH = 1), OD_400nm_ = 0.94, λ_ex_ = 400 nm, and E = 0.9 µJ. Grey bar covers the scattered light from the excitation pulse.

**Figure A9:**
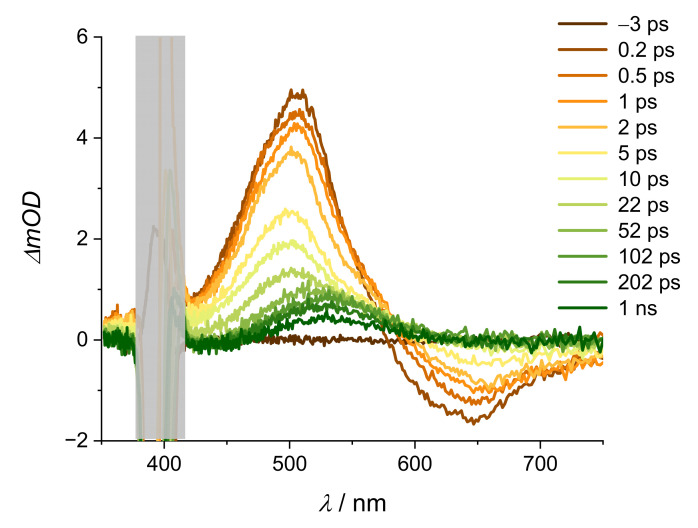
Transient absorption spectra of Na(ARS) solution in water (pH = 3), OD_400nm_ = 0.88, λ_ex_ = 400 nm, and E = 0.9 µJ. Grey bar covers the scattered light from the excitation pulse.

Despite the low fluorescence quantum yields ~1% [2] of the alizarin derivatives, an emission band at 650 nm appears in all transient spectra but without shifting the absorption band. This can be explained by the ESIPT and the respective tautomer. The emission of the tautomer shows a strong bathochromic shift compared to the normal excited form. While the emission of the normal form is dominated by the ESA in the excited state, the emission of the tautomeric form is most likely detected without overlapping processes. Despite the difference in τ_2_, the selected single wavelength transients look quite similar (Figure A7). 

It is important to note that the differences in both systems are small, but this may be mainly due to the advanced dissolution of the [La(OH)_2_][ARS] nanoparticles. Wachtveitl et al. reported an excited state lifetime of 63 ps in methanol [11] after 435 excitation, while Pang et al. excited at 403 and obtained a value 84 ps in ethanol [10]. The time constants (τ_2_) in this work are noticeably lower, therefore, the solvent seems to have a large influence on the lifetime.

## 3. Materials and Methods

### 3.1. Stationary Spectra

Stationary spectra were recorded with a CaryWin500 from Varian (Agilent Technologies, Waldbronn, Germany) at a wavelength interval of 1 nm in a cuvette with a path length of 1 cm and 1 mm, respectively. The spectra shown are corrected for cuvette and solvent effects. 

Fluorescence spectra were recorded with a Fluoromax 4 (Horiba, Oberursel, Germany) with an excitation wavelength of 420 nm and a slit width of 5 nm. The temperature was controlled via a thermostat at 20 °C.

### 3.2. Transient Absorption Spectroscopy

The general procedure for transient absorption spectroscopy has been previously reported [41]. In brief, for detection in the UV–Vis range, a small portion of a Ti:sapphire laser system (Astrella, Coherent, Utrecht, The Netherlands, 800 nm, 1 kHz, 35 fs, 7.2 mJ) was employed to pump a non-collinear optical parametric amplifier (NOPA, Clark-MXR Inc., Dexter, MI, USA). Excitation pulses were generated in the NOPA and by frequency doubling of the fundamental 800 nm pulse in a BBO crystal. Probe pulses between 350 and 750 nm (white light continuum) were generated by irradiating a continuously moving CaF_2_ crystal. This white light was split into two pulses. The first overlapped with the pump pulse in the sample (Starna cuvette, Suprasil quartz, optical path length of 1 mm, continuously stirred by a miniaturized magnetic bar) to monitor pump-induced changes as recorded using a charge-coupled device (CCD) camera (Linescan Series2000, 512 pixels, Si detector, Entwicklungsbüro Stresing, Berlin, Germany). The other part provided a reference pulse, which was detected using an additional CCD camera of the same type. After passing through the sample and before reaching the camera, the white light was dispersed by a prism with an average resolution of roughly 1.5 nm.

Data were processed using an in-house written LabView program. Every second pump pulse was blocked using an optical chopper (Thorlabs, Bergkirchen, Germany), resulting in ΔmOD (10^−3^ ΔOD, where OD represents the optical density, i.e., absorbance) spectra with and without excitation.

### 3.3. Sample Preparation

Alizarin and alizarin red S (ARS) were dissolved in DMSO and diluted with stock solutions of 1,8-Diazabicyclo[5.4.0]undec-7-en (DBU) and toluenesulfonic acid (TosOH), respectively.

The Na(ARS) and [La(OH)_2_][ARS] in water were prepared by Sabljo et al. [9] The particle size of the as-prepared [La(OH)_2_]^+^[ARS]^−^ IOH-NPs was determined by dynamic light scattering (DLS) and scanning electron microscopy (SEM). According to DLS, aqueous suspensions exhibit a mean hydrodynamic diameter of 69 ± 15 nm (Figure 1b). SEM shows a mean diameter of 47 ± 7 nm (Figure 1b,c). The larger particle diameter obtained by DLS reflects the hydrodynamic diameter and the presence of a rigid layer of adsorbed water molecules on the particle surface [9]. The chemical composition of the [La(OH)_2_]^+^[ARS]^−^ IOH-NPs was determined by X-ray diffraction (XRD), Fourier-transformed infrared (FT-IR) spectroscopy, energy dispersive X-ray spectroscopy (EDXS), elemental analysis (EA), and thermogravimetry (TG). Details were published elsewhere [9]. XRD indicates the IOH-NPs to be non-crystalline. FT-IR spectroscopy shows the characteristic vibrations of ARS (*ν*(Ar–C=O): 1700–1600 cm^−1^, *ν*(Ar–C=C): 1590 cm^−1^, *ν*(Ar–C–O): 1260 cm^−1^, *ν_as_*(SO_3_): 1200–1100 cm^−1^, *ν_s_*(SO_3_): 1100–950 cm^−1^, and *ν*(Ar–C–H): 950–600 cm^−1^, Figure 1d). EDXS confirms the presence of lanthanum and sulfur [9]. EA and TG allow us to quantify the chemical composition with a total organics content (TOC) of 59.9% and element contents of 34.1 wt-% C, 2.7 wt-% H, and 6.1 wt-% S (calculated: 59.5% TOC; 34.2 wt-% C, 1.8 wt-% H, 6.5 wt-% S). Taken together, the analytical data prove the cation-to-anion ratio of [La(OH)_2_]^+^:[ARS]^−^ = 1:1 and the composition [La(OH)_2_]^+^[ARS]^−^.

For transient spectroscopy at 400 nm and 530 nm, a concentration of 2.05 µmol/L was used.

## 4. Conclusions

The change from alizarin to ARS allows for the recording of excited state properties in an aqueous environment. Although substantial differences, compared to DMSO, were observed, aqueous solutions of ARS show only small differences when compared to [La(OH)_2_][ARS] nanoparticles. Changing the pH from acidic (pH 3) to neutral (pH 7) leads to significantly different spectral results, in which the nanoparticles show an ultrafast component, indicating partial dissolution. Additional experiments with UV light excitation confirm the findings after excitation in the visible spectrum, and can be advantageous as this does not interfere with the probe pulse detection and shows stronger transient amplitudes. Transient spectra show some differences in dissolved ARS and [La(OH)_2_][ARS] nanoparticles but, all in all, the excited state dynamics remain intact in the nanoparticles. Excitation at 530 nm shows almost similar decay (14 vs. 19 ps). Overall, we submit that [La(OH)_2_][ARS] nanoparticles are good compounds for observing excited state dynamics in aqueous solution, making them suitable biomarkers. Without losing major properties, they can easily be transferred into nanoparticles and retain their spectroscopic signature.

## Figures and Tables

**Table 1:** Time constants at a probe wavelength of 410 nm for Na(ARS) and [La(OH)_2_][ARS] nanoparticles (pH 7) after excitation at 530 nm.

	Na(ARS)	[La(OH)_2_][ARS]
τ_1_/ps	2.1	0.26
τ_2_/ps	19.5	14.2

**Table 2:** Time constants via global fit for ARS (pH 7) after excitation at 267 nm.

	Na(ARS)	[La(OH)_2_][ARS]
τ_1_/ps	6.2	0.8
τ_2_/ps	19.3	6.7
τ_3_/ps	868	1390

**Table 3:** Time constants (global fit) of aqueous Na(ARS) solution and nanoparticles after excitation at 400 nm.

	Na(ARS)	[La(OH)_2_][ARS]
τ_1_/ps	2.9	2.9
τ_2_/ps	26.5	46.8
τ_3_/ps	>>1000	>>1000

**Figure A2:**
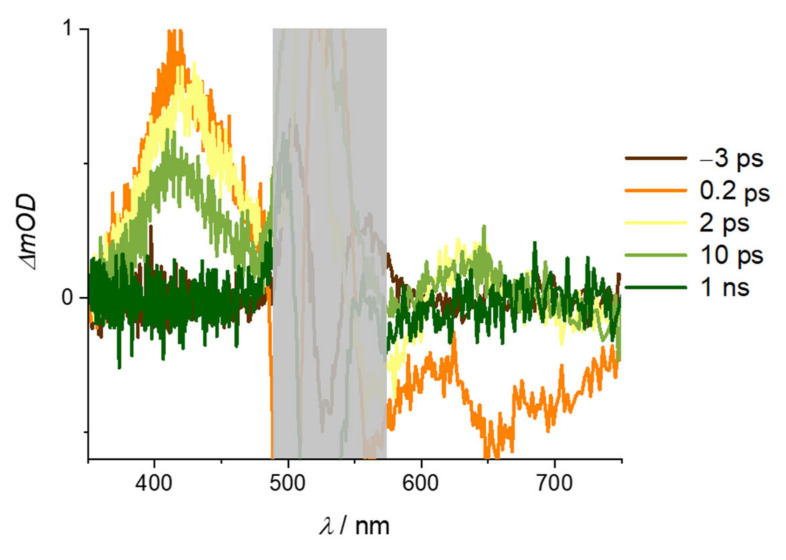
Top: stationary absorption spectrum and bottom: transient absorption spectra of Na(ARS) in H_2_O (pH = 7), OD_530nm_ = 1.62, λ_ex_ = 530 nm, E = 0.9 µJ. Grey bar covers the scattered light from the excitation pulse.

**Figure A10:**
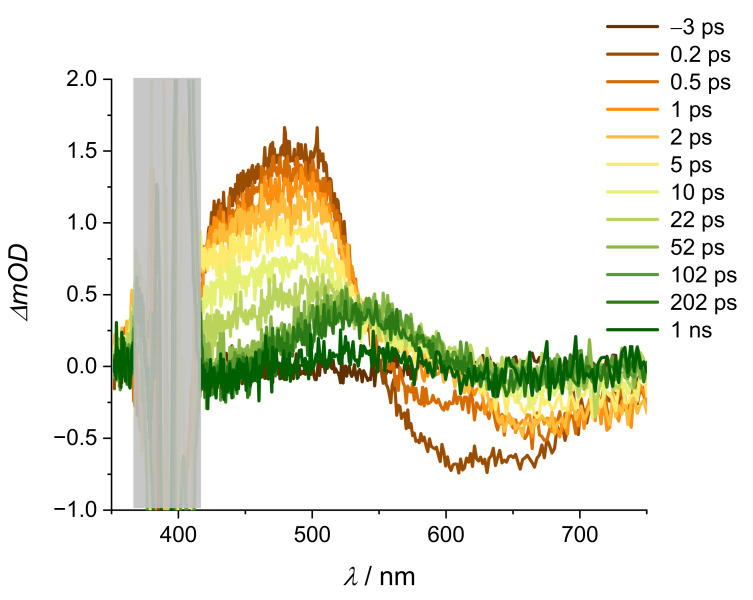
Transient absorption spectra of Na(ARS) solution in water (pH = 5), OD_400nm_ = 0.68, λ_ex_ = 400 nm, and E = 0.9 µJ. Grey bar covers the scattered light from the excitation pulse.

**Figure A11:**
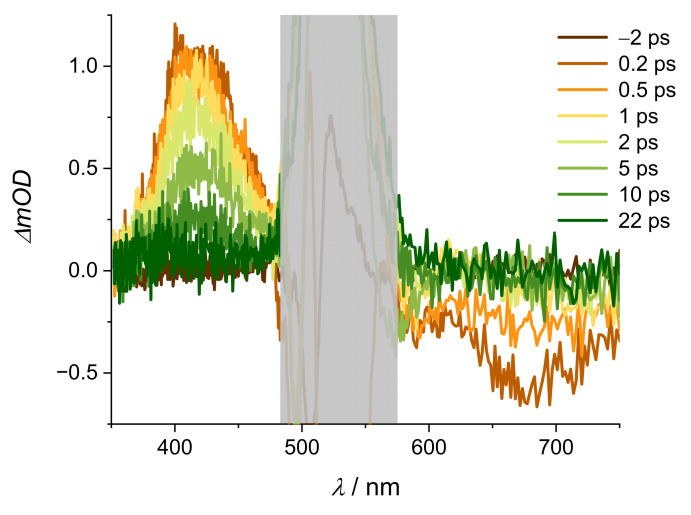
Transient absorption spectra of Na(ARS) solution in water (pH = 10), OD_530nm_ = 1.89, λ_ex_ = 530 nm, and E = 0.9 µJ. Grey bar covers the scattered light from the excitation pulse.

**Figure A12:**
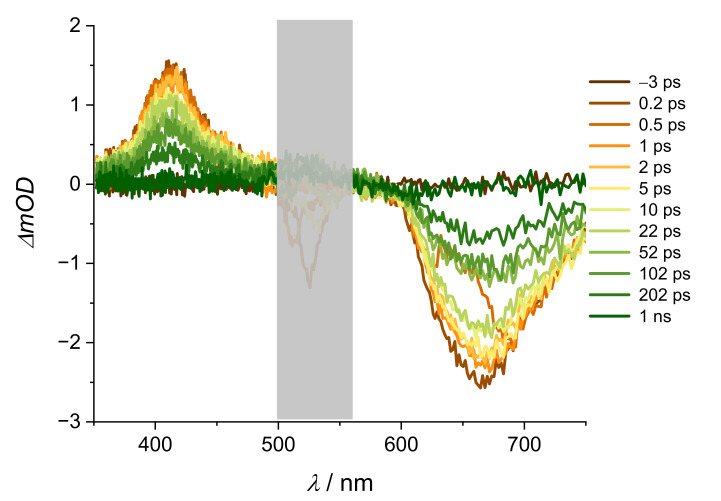
Transient absorption spectra of Na(ARS) solution in water (pH 13), OD_530nm_ = 2.82, λ_ex_ = 530 nm, and E = 0.9 µJ. Grey bar covers the scattered light from the excitation pulse.

**Figure A13:**
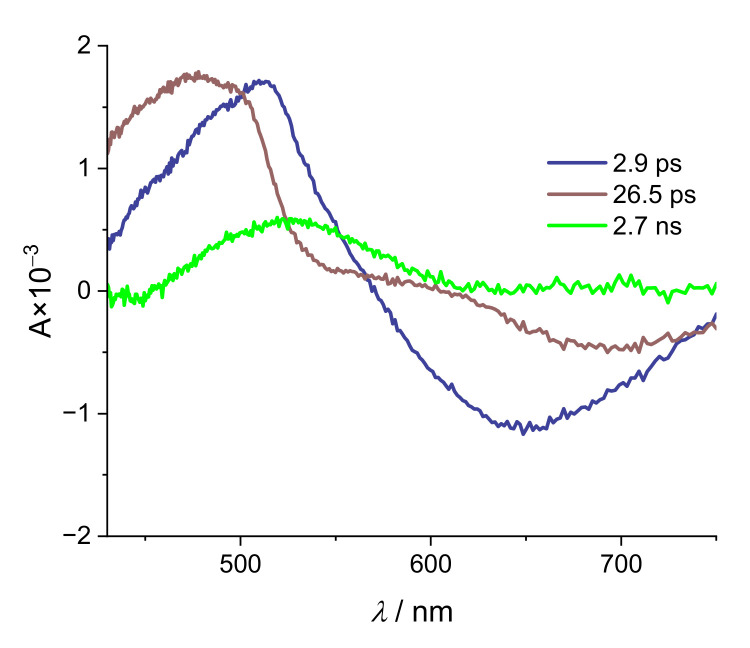
DADS of ARS in H_2_O at pH 3.

**Figure A14:**
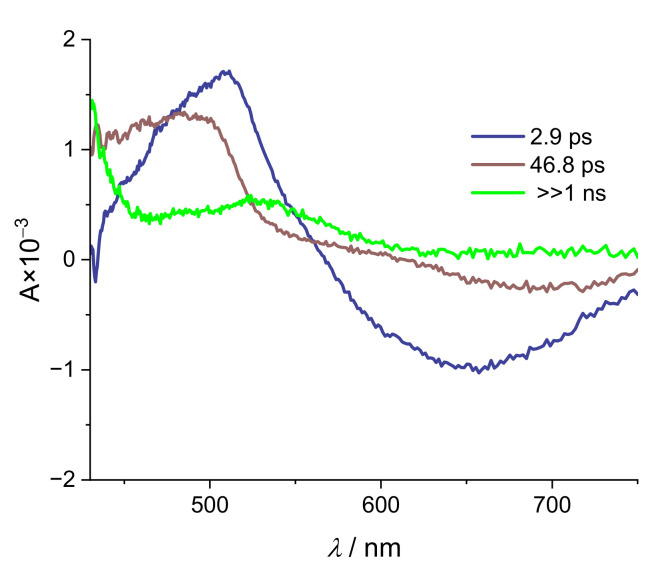
DADS of [La(OH)_2_][ARS] nanoparticles in H_2_O at pH 3.

**Figure A15:**
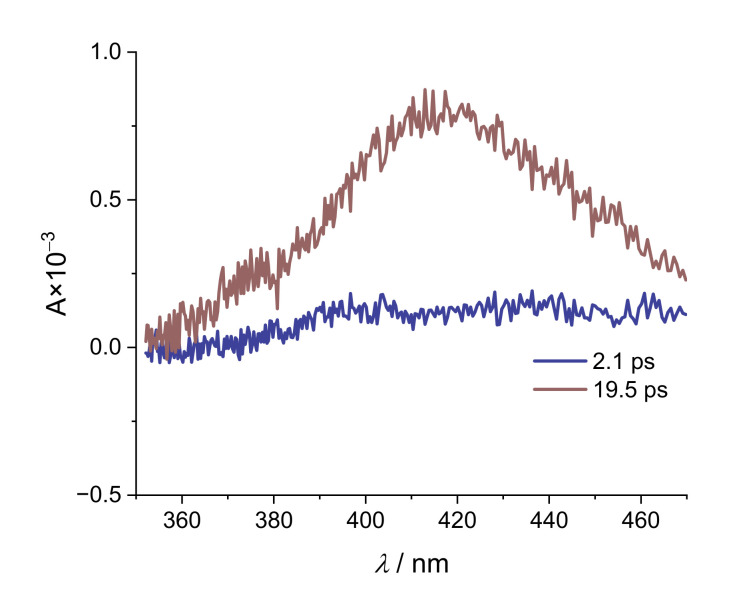
DADS of ARS in H_2_O at pH 7.

**Figure A16:**
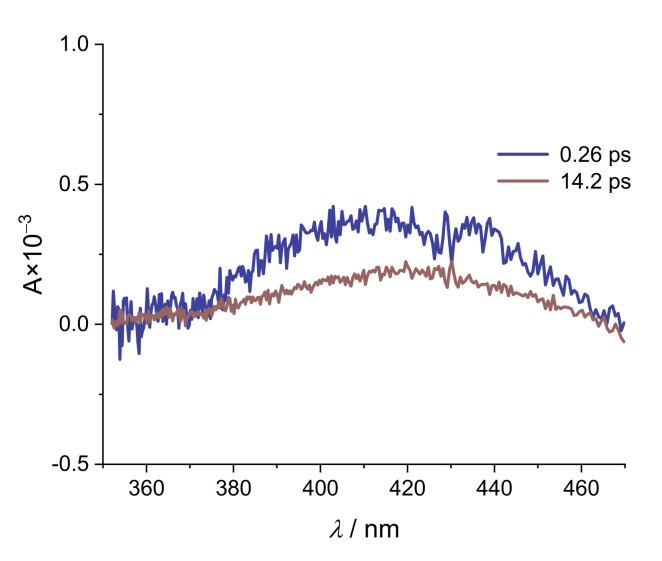
DADS of [La(OH)_2_][ARS] nanoparticles in H_2_O at pH 7.

**Figure A17:**
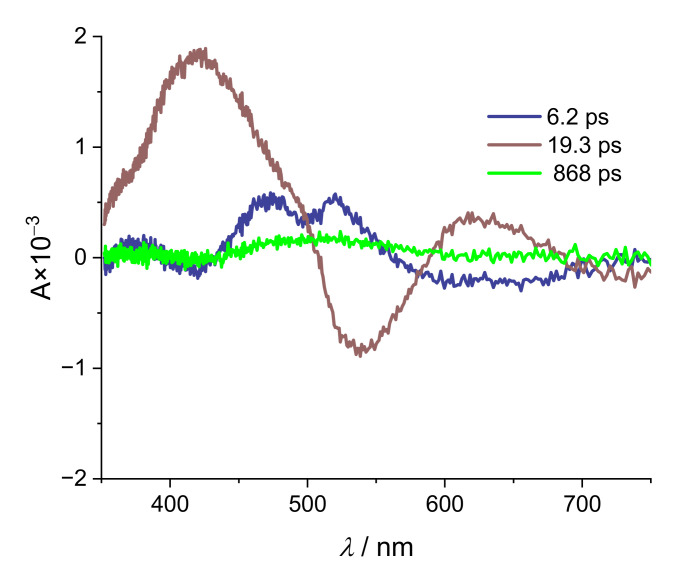
DADS of ARS in H_2_O at pH 7 after UV excitation (267 nm).

**Figure A18:**
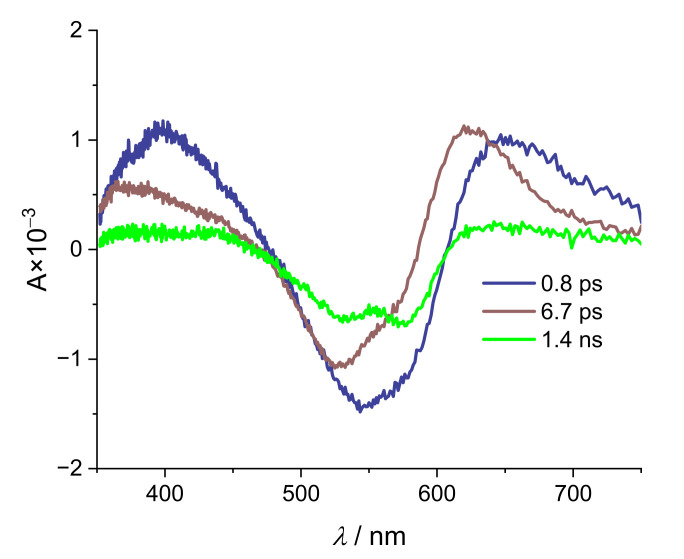
DADS of [La(OH)_2_][ARS] nanoparticles in H_2_O pH 7 after UV excitation (267 nm).

## Data Availability

Datasets generated during the current study are available from the corresponding author upon reasonable request.

## Sample Availability

Samples of the compound [La(OH)_2_][ARS] are available from the authors, as long as stocked.

## Appendix A

**Table A1:** Time constants of Alizarin and ARS in DMSO via global fitting.

	Alizarin/DMSO	ARS/DMSO
τ_1_/ps	0.06	0.1
τ_2_/ps	89	169

**Table A2:** Energy dependent early time constants in acidic ARS.

	1 μJ	1.2 μJ	2.4 μJ
τ_1_/ps	2.9	4.0	3.4
τ_2_/ps	26.5	18.7	24.4
